# The Redox Function of APE1 Is Involved in the Differentiation Process of Stem Cells toward a Neuronal Cell Fate

**DOI:** 10.1371/journal.pone.0089232

**Published:** 2014-02-19

**Authors:** Rossana Domenis, Natascha Bergamin, Giuseppe Gianfranceschi, Carlo Vascotto, Milena Romanello, Silvia Rigo, Giovanna Vagnarelli, Massimo Faggiani, Piercamillo Parodi, Mark R. Kelley, Carlo Alberto Beltrami, Daniela Cesselli, Gianluca Tell, Antonio Paolo Beltrami

**Affiliations:** 1 Department of Medical and Biological Sciences, University of Udine, Udine, Italy; 2 Department of Experimental and Clinical Medical Sciences, University of Udine, Udine, Italy; 3 Department of Pediatrics (Section of Hematology/Oncology), Herman B. Wells Center for Pediatric Research, Indiana University School of Medicine, Indianapolis, Indiana, United States of America; University College London, United Kingdom

## Abstract

Low-to-moderate levels of reactive oxygen species (ROS) govern different steps of neurogenesis *via* molecular pathways that have been decrypted only partially. Although it has been postulated that redox-sensitive molecules are involved in neuronal differentiation, the molecular bases for this process have not been elucidated yet. The aim of this work was therefore to study the role played by the redox-sensitive, multifunctional protein APE1/Ref-1 (APE1) in the differentiation process of human adipose tissue-derived multipotent adult stem cells (hAT-MASC) and embryonic carcinoma stem cells (EC) towards a neuronal phenotype. Methods and results: Applying a definite protocol, hAT-MASC can adopt a neural fate. During this maturation process, differentiating cells significantly increase their intracellular Reactive Oxygen Species (ROS) levels and increase the APE1 nuclear fraction bound to chromatin. This latter event is paralleled by the increase of nuclear NF-κB, a transcription factor regulated by APE1 in a redox-dependent fashion. Importantly, the addition of the antioxidant N-acetyl cysteine (NAC) to the differentiation medium partially prevents the nuclear accumulation of APE1, increasing the neuronal differentiation of hAT-MASC. To investigate the involvement of APE1 in the differentiation process, we employed E3330, a specific inhibitor of the APE1 redox function. The addition of E3330, either to the neurogenic embryonic carcinoma cell line NT2-D1or to hAT-MASC, increases the differentiation of stem cells towards a neural phenotype, biasing the differentiation towards specific subtypes, such as dopaminergic cells. In conclusion, during the differentiation process of stem cells towards a neuroectodermic phenotype, APE1 is recruited, in a ROS-dependent manner, to the chromatin. This event is associated with an inhibitory effect of APE1 on neurogenesis that may be reversed by E3330. Therefore, E3330 may be employed both to boost neural differentiation and to bias the differentiation potential of stem cells towards specific neuronal subtypes. These findings provide a molecular basis for the redox-mediated hypothesis of neuronal differentiation program.

## Introduction

APE1/Ref-1 (Apurinic apyrimidinic Endonuclease/Redox effector factor 1, also called APEX1 or Ref-1 and here referred to as APE1) the mammalian ortholog of *E. coli* Xth (Exo III), is a master regulator of cellular response to oxidative stress and plays a central role in the maintenance of genome stability and transcriptional regulation. Upon removal of the damaged base, APE1 cleaves the abasic site to facilitate DNA repair. The vital effects of APE1 appear to depend on its role in the base excision repair pathways of DNA lesions [1]. However, APE1 also has another major cellular function, since it works as a reduction-oxidation (redox) factor and stimulates the DNA binding activity of several transcription factors that are involved in cell proliferation and differentiation. This function is accounted for by the redox sensitive Cys65. This effect is obtained as a redox co-activation of different transcription factors both involved in cellular response to oxidative stress, such as Nuclear Factor-kappaB (NF-kB), Early growth response protein-1 (Egr-1), p53, Hypoxia-inducible factor 1-alpha (HIF-1α), cAMP response element-binding protein (CREB), activator protein 1 (AP-1) and in differentiation programs such as Paired box containing proteins (Pax) in different cell systems [2]. Recent *in vitro* studies showed that APE1 adopts different unfolded conformations depending on the redox state of its Cys residues, in particular C65 and C93 [3]; moreover, the APE1 redox inhibitor (E)-3-(2-(5,6-dimethoxy-3-methyl-1,4-benzoquinonyl))-2-nonyl propenoic acid (E3330) was shown to decrease the amount of the redox-active protein by driving C65 into disulfide bonds. E3330 holds clinical potential as a specific inhibitor of APE1 redox function, without interfering with its endonuclease activity (for reviews see [4], [5]). The importance of this function is highlighted by results demonstrating that NF-kB-mediated gene expression is regulated by APE1 redox activity, without effects on IκBα degradation [6], [7]. E3330 was also found to selectively inhibit growth/migration of human pancreatic cancer cells [8], suggesting that the APE1 redox function could represent a good candidate for inhibition of tumor invasion and metastasis. We recently demonstrated that E3330-treatment inhibits the TNFα-induced IL8 production driven by NF-κB, in hepatic cancer cell lines [9]. However, knowledge on the detailed molecular mechanisms responsible for the C65-mediated APE1 redox function and for the effects of E3330 inhibition on APE1 *in vivo* are only at its beginning. We recently provided evidence that this redox regulation of APE1 may impact on protein subcellular mitochondrial trafficking [10]. In this regard, the specific block of APE1 redox activity on NF-kB with E3330 impairs hemangioblast development *in vitro* [*11*], thus confirming the leading role of APE1 redox function in affecting the cell differentiation programs. A third non-canonical and poorly characterized APE1 function is represented by its transcriptional activity mediated by direct binding of APE1 to the negative calcium response elements (nCaRE) [12], [13] present on different promoters, including parathormone (PTH), APE1, Bcl-2-associated X protein (Bax) and sirtuin 1 (SIRT1)^10, 11 (Antoniali et al., MBoC in Press)^. The two major functions of APE1, redox and base-excision repair, are completely independent. In fact, the N-terminus, which contains the nuclear localization signal sequence (NLS), is principally devoted to redox-mediated transcriptional co-activation activity and promotes, through its lysine residues, the ability of APE1 to interact both with nucleic acids and with nucleophosmin [14], while the C-terminus exerts the enzymatic activity on the abasic sites of DNA mainly through the residue H309 in the catalytic site [15]. Furthermore, a new unsuspected function of APE1 in RNA metabolism, which is controlled by the N-terminal domain of the protein, has been recently discovered. In particular, it has been demonstrated that APE1 acts as a cleansing factor of abasic rRNA and is able to bind hairpin structures of RNA molecules [16], [17].

APE1 is essential for cell viability [18] and therefore a detailed comprehension of the molecular targets of APE1 functions has been very difficult. Conditional knock-out and knock-down strategies [1], [19] confirmed the essentiality of this protein and allowed establishment of cell models to inspect and characterize, in better detail, the major functions of APE1. However, knowledge of the molecular effectors regulated by APE1 in determining its biological essentiality is still scanty.

Concerning the role played by APE1 on neuronal cells, it seems to be essential both in protecting cells toward oxidative stress and in controlling the differentiation program. In fact, it has been demonstrated that APE1 promotes NF-kB-driven glial cell-derived neurotrophic factor (GDNF) receptor α1 expression, thus inducing GDNF responsiveness, which subsequently both stimulates neurite outgrowth and protects cells from amyloid peptide and oxidative stress [20]. GDNF was originally characterized as a potent neurotrophic factor, specific for the survival and differentiation of the midbrain dopaminergic neurons [21]. Interestingly, APE1 is highly expressed, *in vivo*, in selected regions of the central nervous system [22], [23] supporting its pivotal role for neuronal cells. In pathological settings, a reduction in APE1 expression has been shown to occur: in the hippocampus after hypoxic/ischemic injury [24], in the cortex after compression injury [25], and in the spinal cord after ischemia [26], while increased nuclear levels have been described in Alzheimer’s disease cerebral cortex, corroborating the view that the cellular adaptive response to the oxidative stress condition is involved in the pathogenesis of this disease [27].

However, the role played by APE1 in the differentiation of either embryonic or adult stem cells towards the neural and glial lineages has not been investigated thus far. To this aim, we employed two distinct cell systems: 1) a human Embryonic Carcinoma cell line (hEC) that possesses a well-established ability to differentiate towards a neural fate (NTERA2 cl.D1 -NT2-D1-) [28] and 2) non-immortalized human adult stem cells. Specifically, we isolated and cultured Multipotent Adult Stem Cells (MASC) from adipose tissue, modifying a culture protocol that we already used to isolate similar cells from adult human bone marrow, heart, liver and skin biopsies [29]–[31]. MASC display clonogenicity, self-renewal ability, and multipotency, express pluripotent state specific transcription factors (i.e. OCT4, Nanog, Sox2, and Rex1), display high levels of telomerase activity, and a gene expression profile highly similar, irrespectively from the tissue of origin [29]. Most importantly, MASC can differentiate into neuron-like cells that display, on top of markers of cell differentiation, functional properties of neuronal cells [29].

The goal of the present study was to evaluate the potential role played by the APE1 redox function in the differentiation process of stem cells towards a neuronal fate.

## Materials and Methods

### Tissue Donors and Ethical Approval

Human adipose tissue samples of healthy donors (*n = *31) undergoing plastic surgery were collected after informed consent. The study was approved by the Ethics Committee of Udine (reference number 47831) and a written consent was obtained from each enrolled subject.

### Culture and Differentiation of NT2-D1

NTERA-2 cl.D1 (NT2-D1) cells were purchased from Istituto Zooprofilattico di Lombardia ed Emilia (IZSLER) cultured in Dulbecco’s modified Eagle’s medium (high glucose) containing 10% fetal bovine serum (FBS), 4 mM glutamine and 1% Penicillin/Streptomycin. For neuronal differentiation, NT2-D1 cells were cultured for four weeks in Opti-MEM containing 4% FBS, 4 mM glutamine, 56 µM β-mercapto-ethanol, 1% Penicillin/Streptomycin and 100 mM all-trans retinoic acid (ATRA). Following ATRA treatment cells were split 1∶6. After 1–2 days cultures were mechanically shaken to dislodge cells and these free-floating cells were plated onto Matrigel (50 µg/ml) coated growth surface and treated for two weeks with 10 µM fluorodeoxyuridine, 10 µM uridine and 1 µM cytosine arabinoside. For the inhibition of APE1, E3330 [20 µM] was added to the culture medium starting from the third week of differentiation.

### Human Adipose Tissue Derived MASCs (hAT-MASCs)

After tumescent liposuction, lipoaspirates were centrifuged at 3×10^3^ *g* for 3 minutes and the stromal vascular fraction was collected in a sterile container. The samples were enzymatically dissociated in a 0.05% Collagenase type II solution (Sigma-Aldrich) in Joklik modified Eagle’s Medium (Sigma-Aldrich) for 20 minutes at 37°C. Collagenase activity was stopped by the addition of 0.1% BSA (Sigma-Aldrich) solution in Joklik modified Eagle’s Medium (Sigma-Aldrich). Cell suspension was centrifuged at 1×10^3^ *g* for 10 minutes. Samples collected from different subjects were kept distinct and used to obtain distinct hAT-MASC cell lines.

2.0×10^6^ freshly isolated human cells were plated onto 100 mm human fibronectin (Sigma-Aldrich) coated dishes (BD Falcon) in an expansion medium composed as follows: 60% low glucose DMEM (Invitrogen), 40% MCDB-201, 1 mg/mL linoleic acid-BSA, 10^−9 ^M dexamethasone, 10^−4 ^M ascorbic acid-2 phosphate, 1X insulin-transferrin-sodium selenite (all from Sigma-Aldrich), 2% fetal bovine serum (StemCell Technologies), 10 ng/ml human or murine PDGF-BB, 10 ng/ml human or murine EGF (both from Peprotech EC). Medium was replaced with fresh one every 4 days. Once cells reached 70–80% of confluence, they were detached with 0.25% trypsin-EDTA (Sigma-Aldrich) and re-plated at a density of 1–2×10^3^/cm^2^.

### Flow Cytometry

At passage 3 (P3), cells grown in expansion medium were detached with 0.25% trypsin-EDTA (Sigma-Aldrich) and, after a 20 minutes recovery phase, were incubated with the following properly conjugated primary antibodies: CD10, CD13, CD29, CD49a, CD49b, CD49d, CD90, CD73, CD44, CD59, CD45, HLA-DR, CD117, CD34, CD 271 (BD Biosciences), CD105, CD66e, KDR (Serotech), CD133 (Miltenyi Biotec), CXCR4 (R&D), ABCG-2 (Chemicon International).

### Single Cell Cloning

P2 cells were individually deposited onto the wells of a 96wells Terasaki plate with an automated cell sorter (MoFlo, DakoCytomation) and cultured in expansion medium, as in [29]. About 700 wells were seeded for each analyzed cell line.

### Multilineage Differentiation of hAT-MASC

Multilineage differentiation was induced as in Beltrami et al. 2007.

For neural differentiation, hAT-MASCs were plated at a density of 3×10^3^/cm^2^ in DMEM-HG, 10% FBS, N1. After 24 hours, medium was replaced with a medium added with 1% B27 (Invitrogen), 10 ng/ml EGF (Peprotech) and 20 ng/ml bFGF (Peprotech), N2. After 5 days, cells were washed and incubated for 9 days with DMEM containing 5 µg/ml insulin, 200 µM indomethacin and 0.5 mM IBMX (all from Sigma-Aldrich) in the absence of FBS, N3. For experiments involving the use of antioxidants, 10 mM N-acetyl cysteine was added to the culture medium, while for experiments requiring the inhibition of APE1 redox function, the specific inhibitor E3330 was employed (kindly provided by Prof. M.R. Kelley) [4]. Specifically, in hAT-MASC cultures E3330 was either added at a concentration of 20 µM during the last step of the differentiation protocol (N3) or at a concentration of 40 µM and 20 µM during the last two steps of the differentiation protocol (N2 and N3, respectively).

### Measurement of Intracellular Reactive Oxygen Species (ROS) Generation

Following the manufacturer’s protocol, cells were loaded with 5 µM 5–[and -6]-chloromethyl–2′,7′-dichlorodihydrofluorescein diacetate acetyl ester (CM-H_2_DCFDA, Molecular Probe, Eugene, OR, USA) for 15 minutes, protected from light, in Opti-MEM (Invitrogen). Then, 1 mM H_2_0_2_ was added for 30 minutes as positive control. The cells were washed once with PBS and the production of ROS was visualized by inverted fluorescence microscope and images were quantified by ImageJ software.

### Protein Extraction

To prepare the total protein extracts, 2×10^6^ cells were lysed in 50 mM Tris-HCl pH 7.5; 150 mM NaCl; 1 mM EDTA, 1% Triton X-100 containing a cocktail of protease inhibitor (Sigma-Aldrich), 0.5 mM PMSF, 1 mM NaF, 1 mM Na_3_VO_4_ and 0.1 mM DTT). The suspension was then incubated at 4°C for 30 minutes, sonicated and then subjected to centrifugation for 20 minutes at 12000×g. The supernatant was collected as total extract and stored at −80°C for biochemical assays.

To separate the soluble (S1) and insoluble (P1) fractions cells pellets (3×10^6^cells) were incubated for 10 min at 4°C under orbital rotation in 200 ml ice-cold CSK buffer (100 mM NaCl, 300 mM sucrose, 10 mM PIPES, pH 6.8, 3 mM MgCl_2_, 1 mM DTT, 1 mM EGTA) containing 0.5% Triton X-100 and protease inhibitors. After centrifugation at 5000×g for 5 min, the supernatant (soluble proteins) was recovered (S1 fraction). Pellets were washed three times with 1 ml ice-cold CSK and centrifuged at 5,000×g for 5 min. The resulting pellets (P1, chromatin fraction) were resuspended in 30 ml of 20 mM Tris-HCl buffer, pH 8, 150 mM NaCl, 0.1% NP40, 1 mM EDTA and protease inhibitors. The suspension was sonicated and centrifuged for 30 min at 13,000×g at 4°C and the supernatant was recovered. Protein content was measured using a colorimetric Bradford assay (Bio-Rad Laboratories, Richmond, CA) with bovine serum albumin as a standard.

### Western-blot Analysis

Loading of the extracts was normalized by protein content. Aliquots from the cell extracts were denatured by heating at 95°C for 5 min. Samples were electophoresed on 10% SDS-PAGE and then transferred to nitrocellulose membranes as previously described [32]. To confirm the amounts of protein in each lane, membranes were stained with Ponceau rouge, and a second gel was run in parallel and stained with Comassie. Blots were incubated with the following antibodies: mouse monoclonal anti-APE1 antibody (kindly provided by Prof. M.R. Kelley), and rabbit antibody anti-actin (Sigma) to normalize the protein content in the total extracts; then with the corresponding peroxidase–conjugated anti-serum (Sigma). The bands were visualized and analyzed using a ChemiDoc XRS (Bio-Rad, Milano, Italy) and associated software.

### Immunofluorescence Confocal Analysis

Cells fixed in 4% (wt/vol) paraformaldehyde for 20 min at room temperature were permeabilized for 5 min with PBS–0.25% (wt/vol) Triton X-100 and incubated for 30 min with 5% normal donkey serum in PBS–0.1% (wt/vol) Triton X-100 (blocking solution) to block unspecific binding of the antibodies. Cells were then incubated with the following antibodies: OCT-4, Sox2 and Nanog to evaluate primitive cell transcriptional settings; GATA4, cytokeratin 8-18-19, smooth muscle actin (SMA), connexin43, alfa-sarcomeric actin (ASA), glial fibrillary acidic protein (GFAP), β3 tubulin, Microtubule Associate Protein2 (MAP2), and Basic Myelin Protein to evaluate multilineage differentiation (Table S1). To evaluate the role of APE1 in neuronal differentiation, cells were incubated for 3 h with a mouse monoclonal antibody [32], previously labeled by using the Zenon Mouse IgG Labeling Kit (Molecular Probes), utilizing the Alexa Fluor 488-labeled Fab fragment directed against the Fc portion of the IgG primary anti-APE antibody, in accordance to instructions, diluted 1∶2 in blocking solution alone, or together with anti NFkB p65 rabbit polyclonal antibody 1∶100 (Santa Cruz Biotechnology, CA, USA). In this case, after washing cells were incubated for 90 min with secondary Alexa Fluor 546 conjugated goat anti rabbit antibody. Nuclei were then counterstained by DAPI (Sigma), and the microscope slides mounted and visualized through a Leica TCS SP laser-scanning confocal microscope (Leica Microsystems, Wetzlar, Germany) equipped with a 488-nm argon laser, a 543-nmHeNe laser, and a 63x oil fluorescence objective.

### Quantitative Analysis of Immunofluorescence

To obtain quantitative data of the nuclear levels of APE1, images of immunofluorescently labeled cells were acquired with a fully automated dedicated imaging system (DMI6000B, Leica Microsystems or BD Pathway 850, Becton Dickinson). Scrupulous care was taken to keep constant both the exposure time and gain of the camera, and to set these parameters in order to avoid image saturation. Images of APE1 were taken before acquiring DAPI images and an internal shutter was employed to avoid unnecessary exposure of the specimen to light, thus minimizing photobleaching effects. A quantitation of the total fluorescence of nuclear APE1 staining was obtained employing ImageJ software [33]. A threshold was applied to DAPI images both to measure nuclear areas and to create a mask to measure the average intensity of APE1 fluorescence. APE1 Integrated Fluorescence Intensity (IFI) was computed for each nucleus multiplying each nuclear area for the respective mean grey value of APE1.

### Real-Time RT-PCR

Total RNA was extracted from both non-confluent cultures of undifferentiated and differentiated cells at P3 using the TRIzol Reagent (Invitrogen). After treatment with DNase I (Ambion), first strand cDNA synthesis was performed with 1 µg total RNA using random hexanucleotides and MMLV reverse transcriptase (Invitrogen). Primers were designed from available human sequences using the primer analysis software Primer3 (Table S2). Quantitative RT-PCR was performed using Roche LightCycler 480 Real-Time PCR System and the LightCycler 480 SYBR Green I Master (Roche), following manufacturer’s instructions. HPRT was used as internal control for normalization. LightCycler 480 Basic software (Roche) utilized the second derivative maximum method to identify the crossing point (Cp).

### Statistics

Characteristics of the study population are described using means±SEM. Data were analyzed for normal distribution by Kolmogorov-Smirnov test. T-test or Mann-Whitney test, as appropriate, was used to compare continuous variables between two groups. Drug-treatment assays were analyzed by repeated measurements one-way Anova followed by Bonferroni post-test or by Friedman test followed by Dunn’s post-test, as appropriate. Probability values (p) less than 0.05 were considered significant. Analyses were conducted with Prism, version 4.0c and SPSS20 for Macintosh software.

## Results

### Neuronal Differentiation of EC and Adult Stem Cells

**Figure 1:**
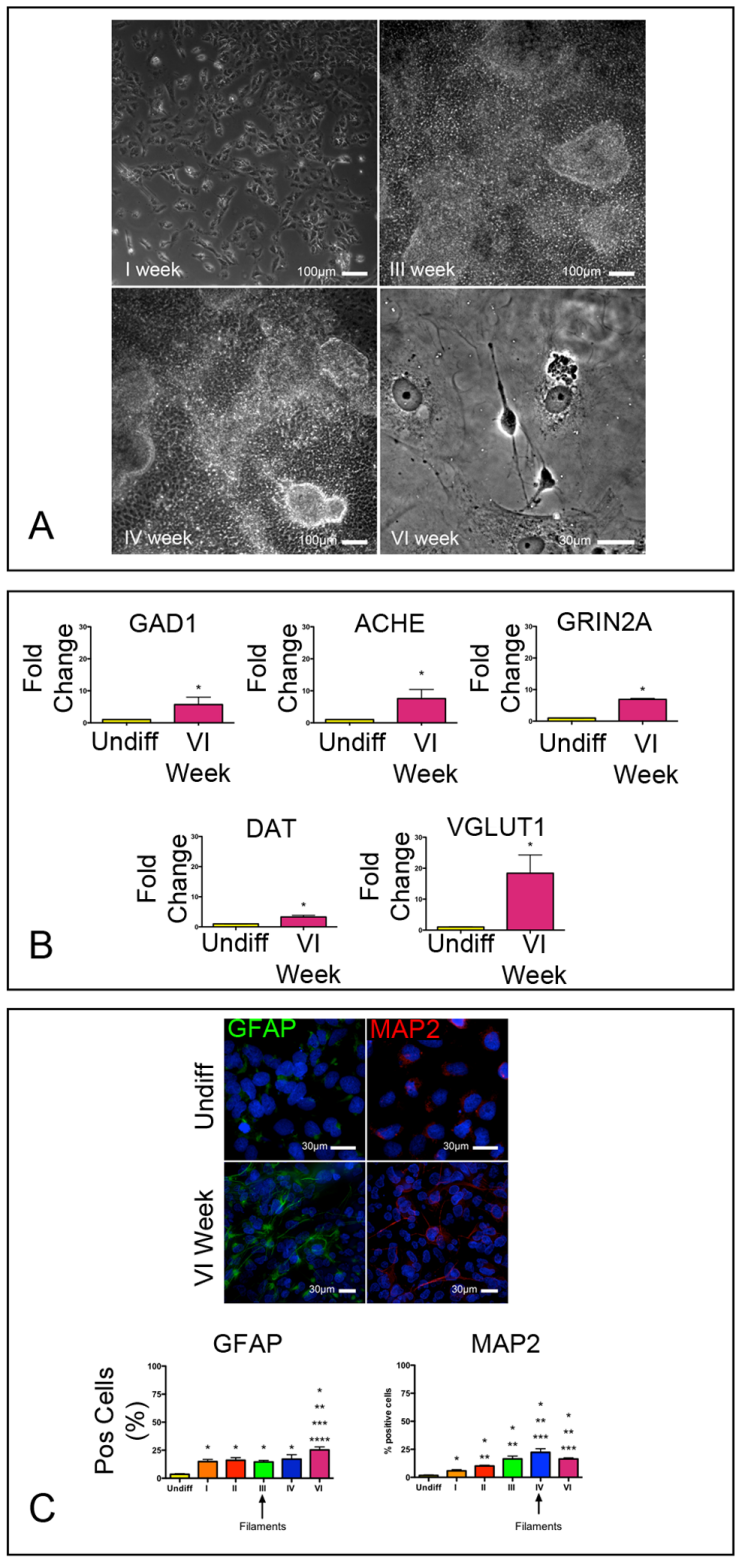
All-Trans Retinoic Acid (ATRA) induces neuronal differentiation of NT2-D1 cells. (**A**) Phase contrast images of NT2-D1 cells exposed to ATRA taken during the 1^st^, 3^rd^, 4^th^ and 6^th^ week of differentiation. (**B**) Histograms showing the relative expression of transcripts of markers of specific neuronal subtypes in differentiated NT2-D1 cells (VI week) versus undifferentiated cells. (**C**) Immunofluorescence images of undifferentiated (upper panels) and NT2-D1 cells exposed to the differentiation protocol for 6 weeks (VI week). GFAP and MAP2 were tested as markers of neural and glial lineages. Histograms represent the fraction (%) of cells positive to the tested markers during the differentiation period. Nuclei are stained in blue by 4′,6-diamidino-2-phenylindole (DAPI). Arrows indicate when, during the differentiation protocol, immunoreactive filaments were first observed. *, **, ***, **** p<0.05 vs I, II, III, and IV column, respectively.

To dissect the role played by APE1 on neuronal differentiation, we first used NT2-D1, a human teratocarcinoma cell line that can generate central nervous system neurons [28]. To induce their differentiation towards a neural fate, we adopted a protocol that involved the use of retinoic acid (ATRA) treatment for four consecutive weeks, followed by the incubation of the cells for two additional weeks in retinoic acid-free medium (Figure 1A). To verify the level of differentiation reached by NT2-D1, cells were characterized at the gene and protein expression levels. After one week of exposure to ATRA, NT2-D1 cells significantly down-regulated Nanog, Nestin and Sox2, representing markers of an undifferentiated state. This latter transcription factor, however, was still expressed at low levels by a large fraction of cells, being typically expressed by neuronal progenitors (Figure S1). Neuronal commitment and differentiation was evaluated by immunofluorescence and by realtime PCR, through which we analyzed transcripts of genes typically expressed by specific cell subtypes (Figure S2). Quantitatively, differentiated cells showed a significant increase in transcripts for glutamate decarboxylase (GAD1, a marker of GABAergic neurons), acetylcholinesterase (ACHE, a marker of cholinergic neurons), dopamine transporter (DAT, expressed by dopaminergic neurons), vesicular glutamate transporter1 (VGLUT1, marker of glutamatergic neurons) and Glutamate receptor subunit epsilon-1 (GRIN2a) (Figure 1B). Concomitantly, NT2-D1 cells significantly increased their positivity to MAP2 and GFAP, known markers of neural and glial cells, respectively. These proteins became organized showing a filamentous pattern starting from the third week of differentiation (Figure 1C).

**Figure 2:**
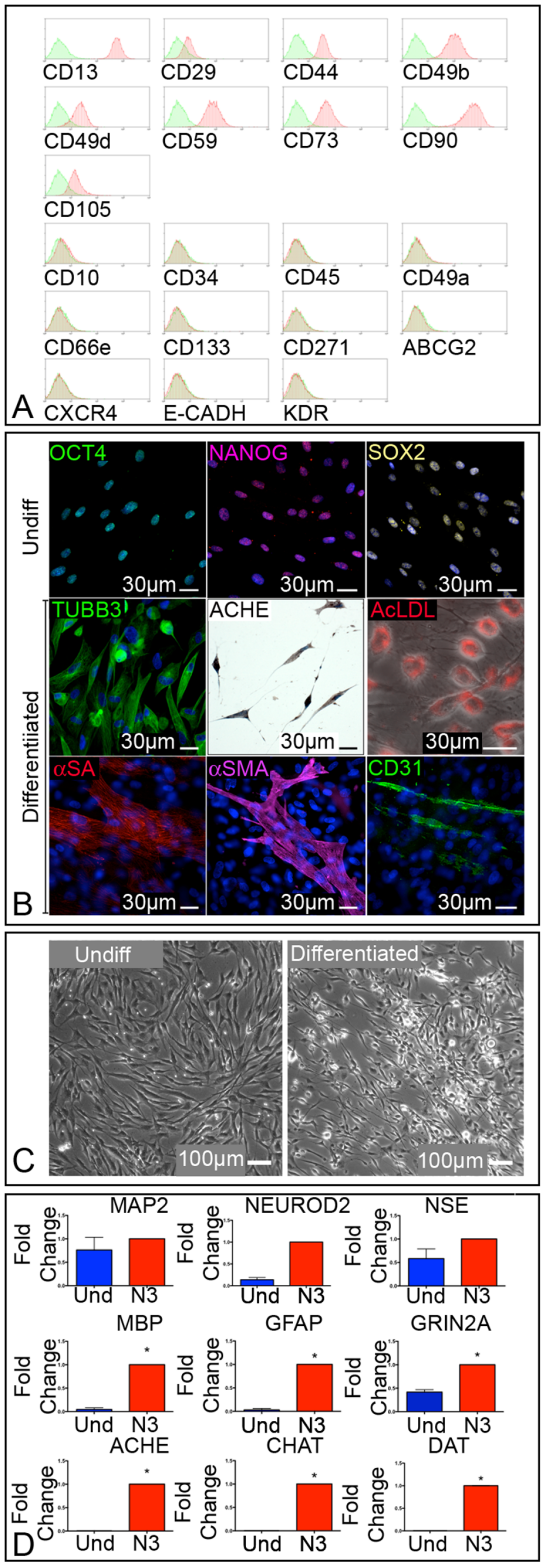
Characterization and neuronal differentiation of human adipose-tissue derived Multipotent Adult Stem Cells (hAT-MASC). (**A**) Representative flow cytometry histograms of a hAT-derived multipotent stem cell population. Plots show isotype control IgG-staining profile (green histogram) versus specific antibody staining profile (red histogram). hAT-MASCs express high levels of mesenchymal stem cell markers (CD90, CD73, CD44, while they are negative for hematopoietic markers CD45 and CD34). (**B**) Immunofluorescence images of undifferentiated and differentiated hAT-MASC. Undifferentiated cells express the pluripotent state specific transcription factors Oct4 (green), Nanog (purple) and Sox2 (yellow). When exposed to neural differentiation medium these cells express the neural markers ß3 tubulin (TUBB3, green) and acetylcholinesterase (ACHE, brown). Once induced to differentiate into hepatocytes, hAT-MASC uptake acetylated LDL (AcLDL, red). Once exposed to myogenic or endothelial media, these cells express either alpha-sarcomeric actin (ASA, red), smooth muscle actin (SMA, purple) or PE-CAM (CD31, green). Nuclei are stained by DAPI (blue). (**C**) Phase contrast images of undifferentiated and differentiated hAT-MASC. (**D**) Histograms showing the relative expression of transcripts of neuronal and glial markers in differentiated hAT-MASC cells (N3, red histogram) versus undifferentiated cells (blue histogram).

As a second cellular model, we employed primary human Multipotent Adult Stem Cells isolated from adipose tissue (hAT) samples [31], adapting to this tissue the method that we previously used to generate these cells from human liver, heart, bone marrow, and skin biopsies [29], [30]. Briefly, we generated 31 plastic adherent, proliferating cell lines from adipose tissue samples with very high efficiency. After three passages in culture, hAT-derived cell lines were characterized to check the acquisition of MASC features. Adipose-tissue derived cell lines showed a mesenchymal cell surface immunophenotype (Figure 2A), expressed the transcription factors Oct-4, NANOG and SOX2, were clonogenic and multipotent (Figure 2B). In order to induce hAT-MASC differentiation towards a neuronal fate, a three-step protocol was employed. hAT-MASC were first exposed to a high serum culture medium (N1) for one day, and subsequently to EGF and bFGF for 5 days (N2). At the end of this commitment period, cells were maintained in a serum-free medium containing insulin, indomethacin, and IBMX for 2–5 days (N3). During this last step cells displayed a remarkable morphological change: starting from a flattened and polygonal shape, their cytoplasm retracted towards the nucleus, leaving long cellular processes peripherally (Figure 2C). Quantitatively, differentiated cells showed, with respect to undifferentiated hAT-MASCs (n = 6), a significant increase in transcripts for myelin basic protein (MBP), glial fibrillary acidic protein (GFAP), Grin2A, choline acetyltransferase (CHAT), acetylcholinesterase (ACHE), and dopamine transporter (DAT), suggesting a stochastic maturation of primitive cells towards different neuronal and glial phenotypes (Figure 2D).

Altogether these results demonstrated that we could obtain cells with similar neuronal properties from both adult and EC stem cells.

### Reactive Oxygen Species (ROS)-dependent Nuclear Translocation of APE1 during Neuronal Differentiation

**Figure 3:**
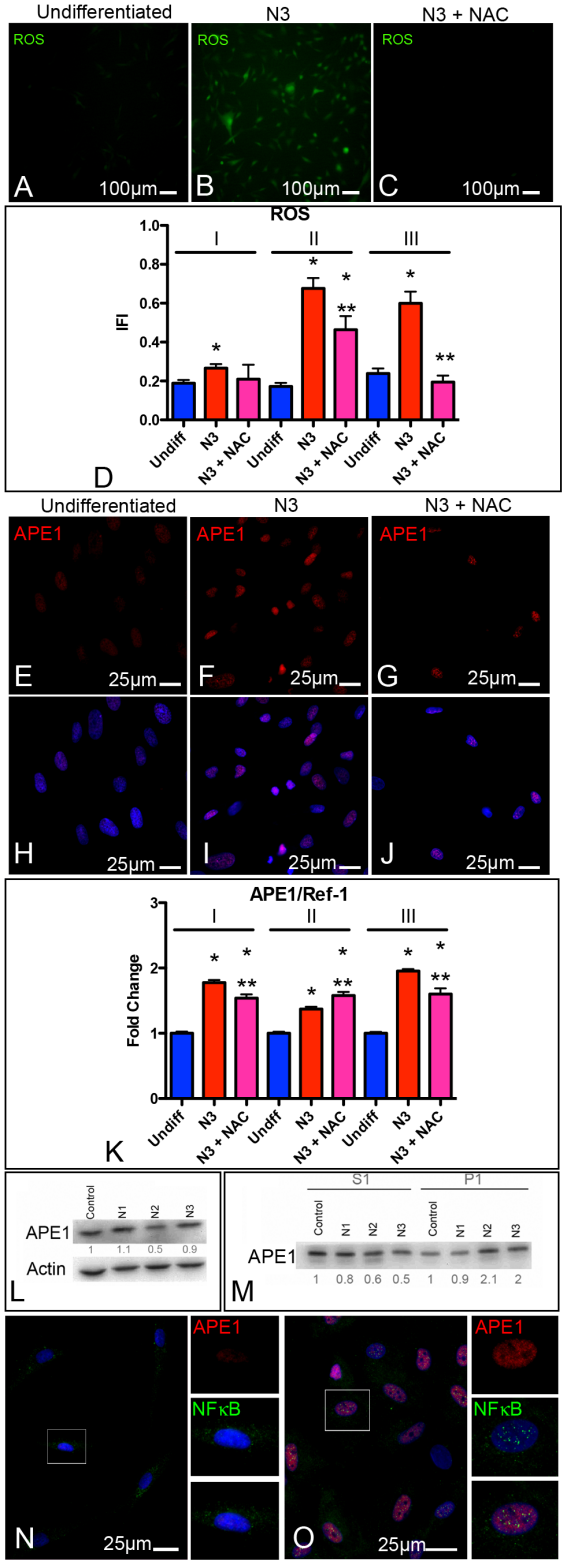
Differentiation towards a neuronal fate increases both intracellular ROS levels and the nuclear localization of APE1. (**A–D**) Epifluorescence images of undifferentiated (undiff, **A**), and differentiated hAT-MASC cultured either in the absence (N3, **B**) or in the presence (N3+NAC, **C**) of NAC. The ROS sensitive, green fluorescent dye CM-H_2_DCFDA was used to quantitate intracellular ROS levels. Histograms (**D**) show the quantification of the Integrated Fluorescence Intensity (IFI) of the above-described groups in 3 (I–III) independent hAT-MASC lines. (**E**–**G**) Immunofluorescence images of APE1 (red) in undifferentiated, N3, and N3+NAC groups. (**H–J**) Nuclear localization of APE1 is shown superimposing to the above images, the blue fluorescence of DAPI staining. APE1 expression was quantified in the three groups by analyzing the IFI of APE1 nuclear fluorescence. Histograms (**K**) summarizing data for APE1 nuclear quantitative fluorescence. (**L**) Representative Western blot of APE1. 10 µg of total protein extracts were loaded onto a 10% SDS-PAGE, blotted and incubated with the APE1 primary antibody and horseradish peroxidase-conjugated secondary antibody. Numbers at the bottom were obtained from densitometric analysis of three independent experiments, normalized versus actin. (**M**) Cells in the different stages of differentiation were separated into fractions S1 (soluble proteins) and P1 (proteins bound to DNA). Equivalent amounts were analyzed by western blot with antibody against APE1. Numbers at the bottom were obtained from densitometric analysis of three independent experiments, normalized versus a gel run in parallel and stained with Comassie. (**N, O**) Immunofluorescence images showing colocalization of APE1 (red) with the p65 subunit of NFkB (green) obtained both in undifferentiated and differentiated (N3) hAT-MASC. Cells comprised in the square are shown at higher magnification in the panels localized at the right side of each picture, where the contribution of APE1 and NFkB are shown as separate images. *, ** p<0.05 vs Undifferentiated or N3 cells, respectively.

With the aim of exploring the involvement of ROS and APE1 in the differentiation of stem cells towards a neuronal fate, we first exposed hAT-MASC to the differentiation protocol and analyzed intracellular ROS levels. As shown in figures 3A–D, intracellular ROS significantly increased upon differentiation. This effect was abrogated, at least in part, by the addition of the ROS scavenger N-acetyl cysteine (NAC) to the culture medium. Quantitative immunofluorescence analysis of APE1 showed a similar trend (Figure 3E–K). Specifically, in non-differentiated cells (from undifferentiated to N2), APE1 was predominantly localized to the nucleus, while there was a significant increase in the nuclear content of the protein in N3, the most differentiated stage. The addition of NAC during the differentiation stage partially abolished this latter event (Figure 3K).

We then tested whether the observed nuclear increase was accompanied by a parallel increase in protein expression. As shown in (Figure 3L), testing APE1 expression in total protein extracts, we were not able to demonstrate the occurrence of any significant increase in total expression concomitant to the proceeding of differentiation. This was confirmed by RT-PCR experiments (data not shown), suggesting that the increase of APE1 in the nucleus was dependent on a nuclear accumulation of the protein rather than on a mere change in the expression levels.

We then analyzed the amount of APE1 bound to insoluble chromatin fraction, by performing a biochemical extraction protocol. The soluble protein fraction (S1) was recovered by extracting cells with CSK buffer. The resulting cell pellet (P1) corresponded to the proteins bound to DNA. Using these extraction conditions, we demonstrated a two fold increase in the expression of APE1 bound to DNA in the N2 and N3 stages compared to the non-differentiated (Control) or proliferating cells (N1) (Figure 3M).

The increase of APE1 bound to chromatin suggested the existence of a correlation with an increase of its DNA-related functions (i.e. both repair and/or transcriptional activities). Therefore, we tested whether this observation could correlate with the functional activity of NF-kB, a well-known transcriptional factor regulated by APE1. As expected, we observed a nuclear accumulation of p65-NF-kB, which correlated with the increase in the intensity of APE1 nuclear fluorescence (Figure 3N, O).

### APE1 Redox Function Drives the Neuronal Fate of Differentiating Stem Cells

Last, we studied the involvement of APE1 in the neuronal differentiation of both adult and EC stem cells.

**Figure 4:**
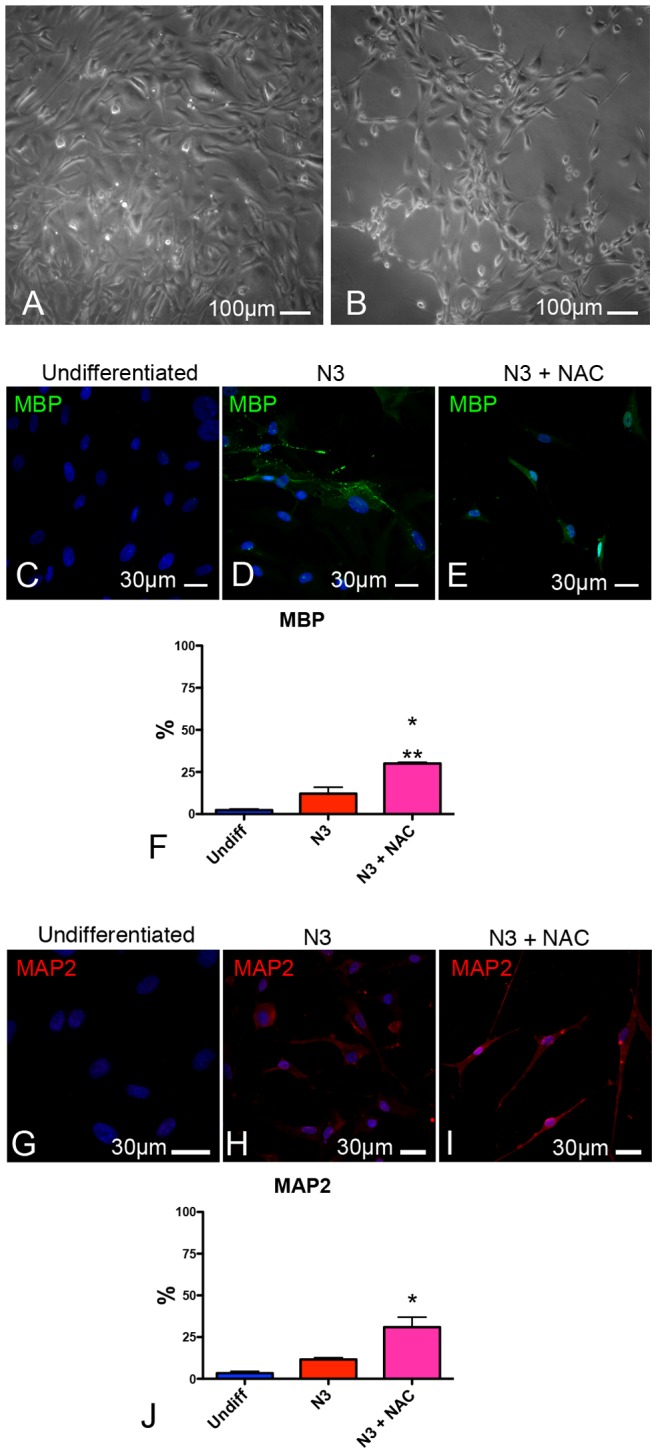
Impact exerted by ROS on the differentiation of adult stem cell towards a neural fate. (**A**, **B**) Contrast phase images of hAT-MASC differentiated in the absence (**A**) or in the presence (**B**) of NAC. Fluorescence images showing MBP (**C**, green) and MAP2 (**G**, red) expression in undifferentiated hAT-MASC and in hAT-MASC differentiated either in the absence (**D**, **H**) or presence of NAC (**E**, **I**). Histograms (**F**, **J**) represent the fraction of cells positive for MBP or MAP2. n = 3 distinct hAT-MASC lines; *, ** p<0.05 vs Undifferentiated or N3 cells, respectively.

For this purpose, we first evaluated the impact exerted by the addition of NAC to the neural differentiation media of hAT-MASC. As shown in figure 4A-B, the morphology of cells reaching the end of the differentiation period was dramatically affected by the antioxidant treatment. Additionally, NAC significantly increased the fraction of differentiated cells expressing the neuronal marker MAP2, without affecting the expression of the glial marker MBP (Figure 4C–J).

**Figure 5:**
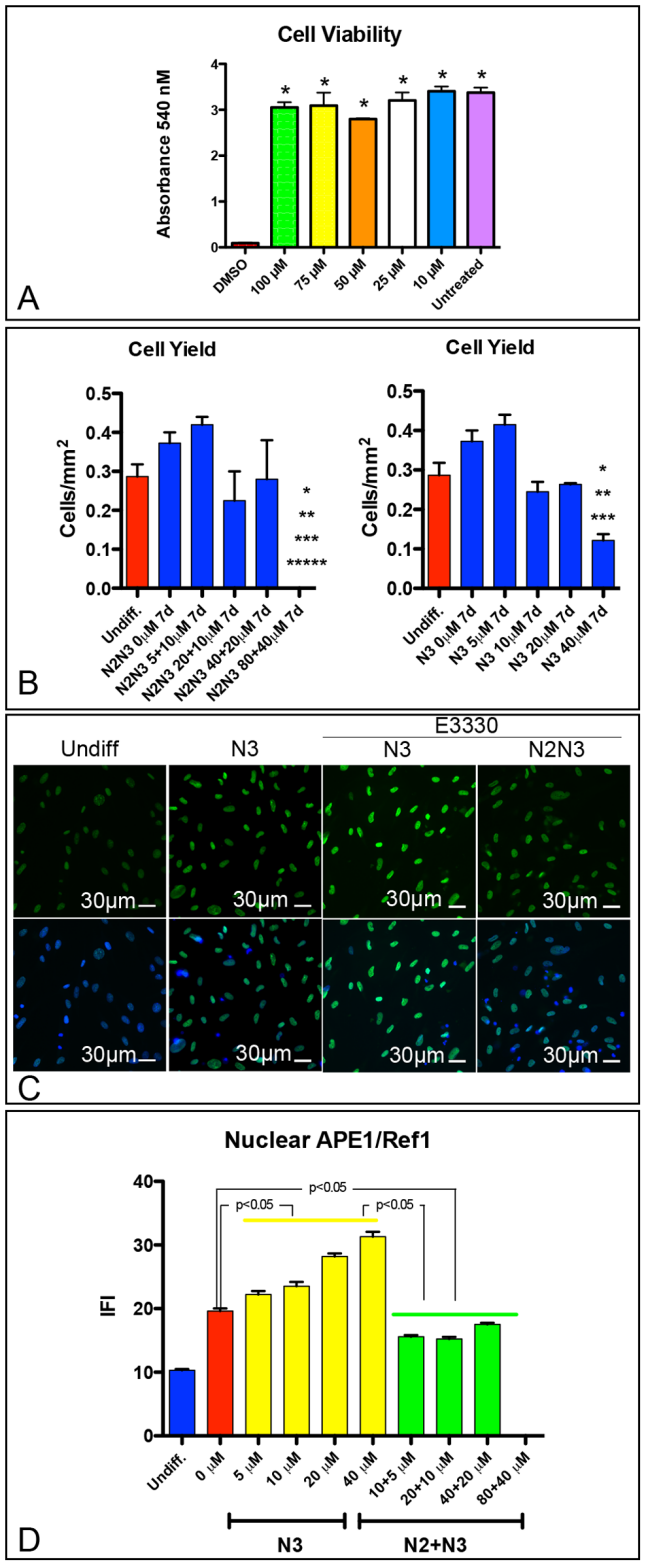
Effects of E3330 on cell viability, proliferation and APE1 expression. (**A**) Histograms summarizing the results of a viability assay (MTT) performed following the exposure of undifferentiated hAT-MASC to either positive control (DMSO), negative control (untreated), or 10 µM, 25 µM, 50 µM, 75 µM, and 100 µM of E3330. (**B**) Histograms summarizing the effects of the exposure of hAT-MASC to E3330 -during either the last step (N3) or the last two steps (N2N3) of differentiation- on the yield of cells at the end of the differentiation protocol. (**C**) Immunofluorescence images showing the effects the exposure of hAT-MASC to E3330–during either N3 or N2N3- on APE1 expression (green). Nuclei are blue labeled by DAPI. (**D**) Quantification of Ape1 nuclear expression (IFI) is summarized in histograms. *, **, ***, ***** p<0.05 vs I, II, III, and V column, respectively.

Based on these data, we investigated the role of APE1 redox function on neuronal differentiation using an APE1 specific redox inhibitor E3330. For this purpose, we first assessed the toxicity of E3330 on hAT-MASCs, exposing these cells either during the last step (N3) or during the last two steps of the differentiation protocol (N2 and N3). E3330, at a dose ranging from 10–100 µM, had no apparent effect on undifferentiated hAT-MASC viability, as assessed by MTT assay (Figure 5A), while its addition to differentiating cells significantly affected cell yield (Figure 5B). Therefore, for subsequent studies, we employed a dose and administration scheme that balanced E3330 toxicity with its effects on APE1. Interestingly, the addition of E3330 during the first day of incubation with N3 medium significantly increased nuclear levels of APE1, as detected by quantitative fluorescence imaging (Figure 5C, D). On the contrary, when E3330 was added during the entire differentiation period, we observed a significant decrease in APE1 nuclear levels (Figure 5C, D).

**Figure 6:**
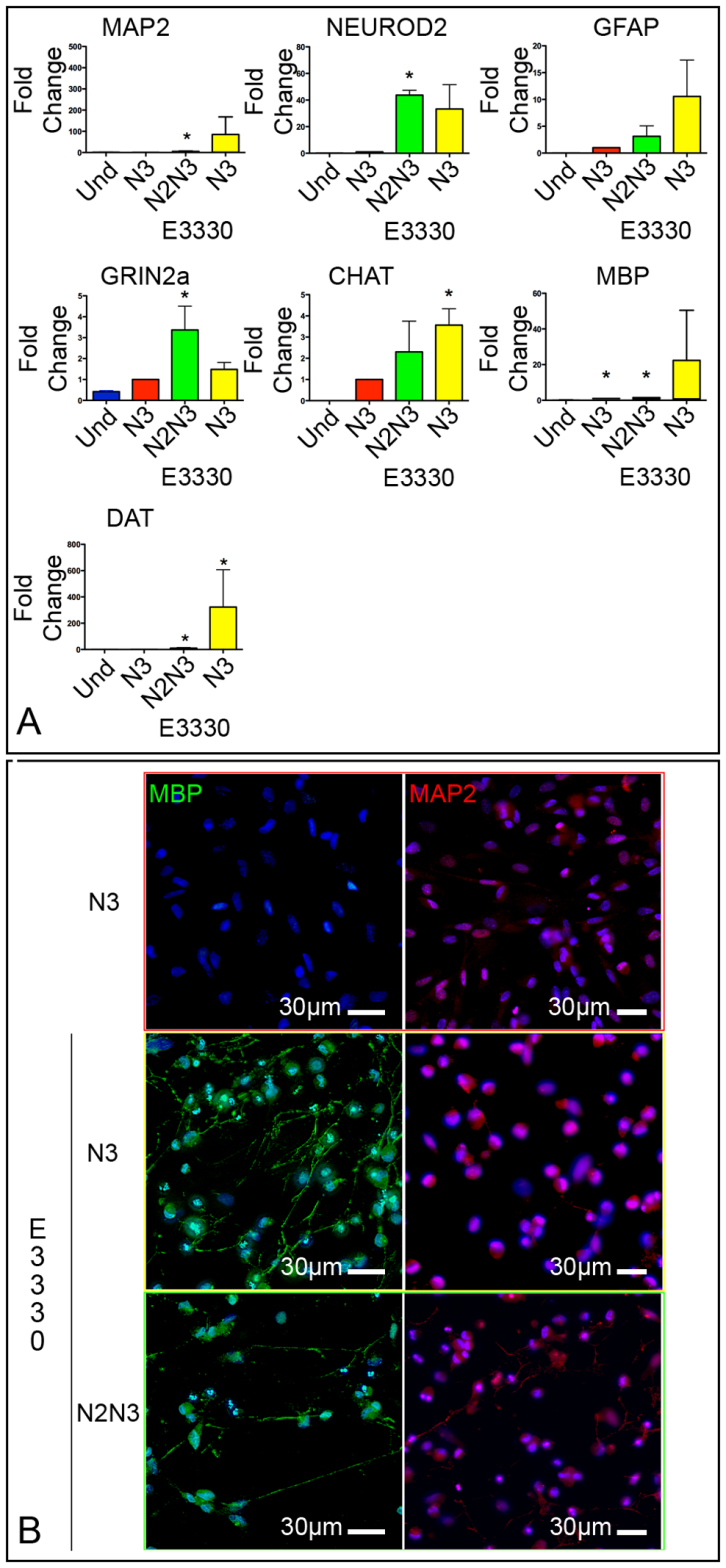
E3330 increases the neuronal differentiation of hAT-MASC. (**A**) Histograms showing the relative expression of transcripts of neuronal and glial markers in undifferentiated and differentiated hAT-MASC, exposed or not to E3330. (**B**) Immunofluorescence images of differentiated hAT-MASC that were either not exposed (upper 2 panels) or exposed (lower 4 panels) to E3330 during the differentiation protocol. Cells were stained for the glial marker MBP (green) and for the neuronal marker MAP2 (red). * p<0.05 vs Undifferentiated cells.

The addition of E3330 during the differentiation protocol, significantly increased transcripts associated with neuronal differentiation; specifically, with respect to differentiated hAT-MASCs not exposed to E3330, the exposed ones up-regulated the transcripts for the neuronal markers NeuroD2 and MAP2 (Figure 6A). Furthermore, E3330-treatment increased the expression of markers of cholinergic (CHAT), and dopaminergic (DAT) neurons (Figure 6A). Last, cells exposed to E3330 showed an increase in immunoreactivity against both MAP2 and MBP (Figure 6B).

**Figure 7:**
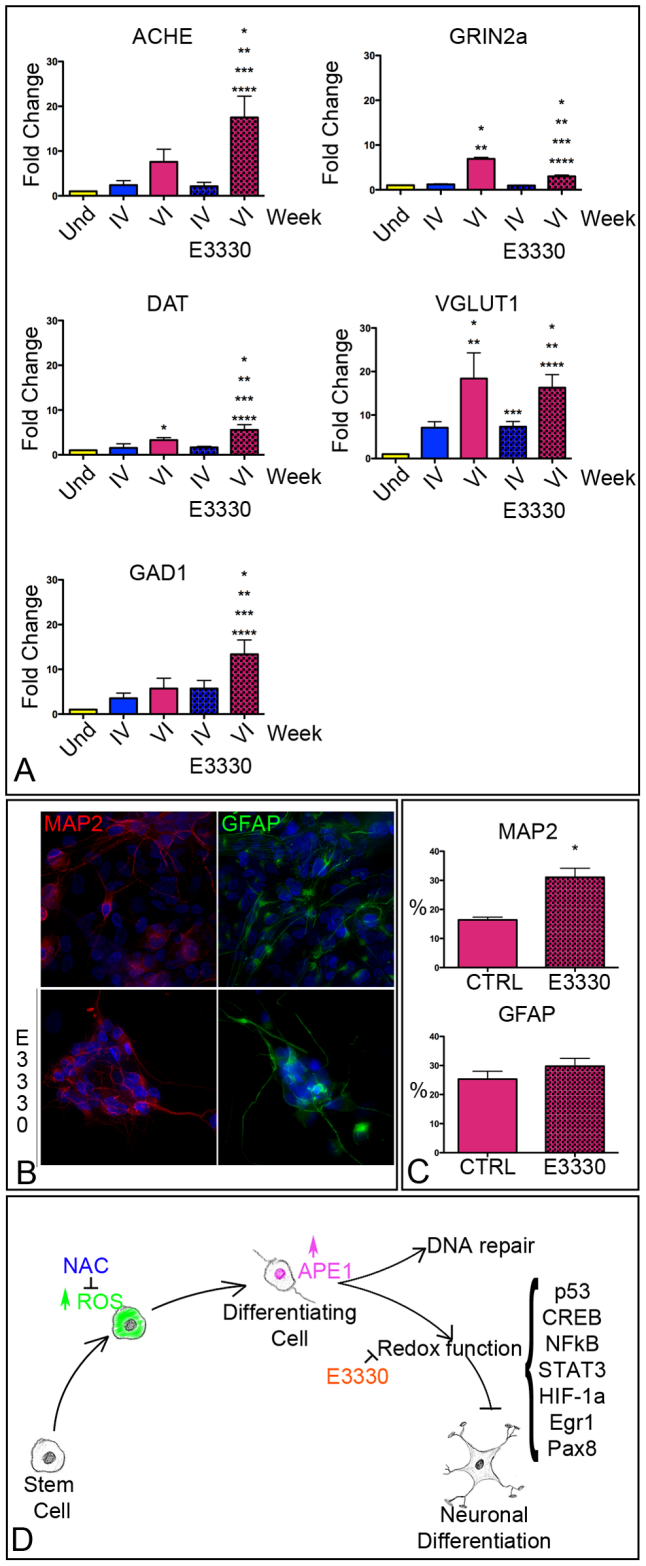
E3330 increases the neuronal differentiation of hAT-MASC. (**A**) Histograms showing the relative expression of transcripts of markers of specific neuronal subtypes in undifferentiated and differentiated NT2-D1, exposed or not to E3330, at the IV and VI week of differentiation. (**B**) Immunofluorescence images of differentiated NT2-D1 that were either not exposed (upper panels) or exposed (lower panels) to E3330 during the differentiation protocol. Cells were stained for the glial marker GFAP (green) and for the neuronal marker MAP2 (red). (**C**) Histograms summarizing the fraction of cells positive to MAP2 and GFAP at the end of the VI week of differentiation in the absence (−E3330) or presence (+E3330) of Ape1 inhibition. (**D**) Diagram summarizing the main findings of this work on the involvement of Ape1 in neuronal differentiation. Arrows with blunt ends indicate inhibition. * p<0.05 vs I column.

Similar results were also obtained exposing the cell line NT2-D1 to E3330 starting from the 3^rd^ week of differentiation. Specifically, with respect to the non-treated ones, E3330-treated cells expressed significantly higher levels of GAD1, ACHE, and DAT. On the contrary, Vesicular glutamate transporter 1, and Grin2A were not up-regulated by the inhibition of APE1 redox function (Figure 7A). Last, E3330-treatment significantly increased the immunoreactivity of differentiated cells against MAP2, while it had no effect on GFAP (Figure 7B, C).

Altogether these results indicate that the redox function of APE1 regulates stem cell differentiation towards a neurogenic fate, reducing it and modulating the differentiation towards specific neuronal subtypes.

## Discussion

This work has several aspects of novelty that have been summarized in Figure 7D: i) APE1 accumulates, in a ROS-dependent fashion, into the nuclei and associates to the chromatin of adult stem cells differentiated towards a neuronal fate, ii) the redox function of APE1 plays a role in the neuronal differentiation of both adult and embryonic stem cells, and iii) the use of a specific inhibitor of the redox function of APE1 increases the extent of neuronal differentiation, facilitating the differentiation towards specific neuronal subsets.

A growing body of literature obtained on different cell models (most of which obtained from rodents) has demonstrated that low-to-moderate levels of ROS govern different steps of neurogenesis, ranging from progenitor cell proliferation to neuronal differentiation [34]. Consistently, we show in the present work that intracellular ROS levels significantly increase during the differentiation process of human AT-MASC towards a neural phenotype. However, we newly demonstrate that the addition of an antioxidant (NAC) to the culture medium of hAT-MASC may alter the extent of differentiation of this cell type. To gain insights into the mechanism involved in this process, we focused our attention on APE1, since Reactive Oxygen Species (ROS) may regulate the activity of this multifunctional protein both at the transcriptional level and at the post-translational one, promoting its cytoplasmic to nuclear translocation [2]. This latter event, which is regulated by acetylation of conserved K residues present in the N-terminal domain of APE1, has been associated to the functional activation of several target transcription factors [35]. In line, we found that hAT-MASC exposed to a neural differentiation protocol are characterized by a progressive nuclear accumulation of both APE1 and one of its target transcription factors (i.e. nuclear factor-kB, NF-kB). These events occur in the absence of a net increase in the total levels of APE1 and are paralleled by a progressive increase in the abundance of chromatin-bound APE1. Importantly, NAC can partially reduce the nuclear accumulation of APE1. Altogether our results support the hypothesized role played by redox-sensitive transcription factors on neurogenesis [34].

Given the complete independence of the two major functions of APE1 (i.e. the repair function and the redox one) [36], we took advantage of the ability of the drug E3330 to inhibit selectively this latter, without affecting the first one. Our results demonstrate that E3330, at a dose ranging from 10–100 µM, is not toxic, when given to undifferentiated cells. However, the addition of this compound to the differentiation media of either hAT-MASC or NT2-D1 significantly increases, in both cell types, the expression of markers of neuronal differentiation (e.g. MAP2). Altogether these results suggest that the redox function of APE1 exerts an inhibitory effect on the maturation of both adult and embryonic human stem cells towards the neuronal fate. Consistently, several transcription factors that are regulated by APE1 in a redox-dependent manner have been implicated in neurogenesis (e.g. p53, CREB, NF-kB, STAT3, HIF-1α, Egr1, and Pax8). Among these, a primary inhibitory role on neural differentiation is played by p53. In fact, p53 deficiency leads to increased neurogenesis, biased differentiation *in vivo* and an incremented *ex vivo* proliferation of neural stem cells [37], [38]. STAT3 too may bias stem cell differentiation, reducing neurogenesis, as shown by the observation that neural precursor cells differentiate into astrocytes after stimulation with CNTF or LIF via STAT3 activation [39], [40].

Last, the addition of E3330 to differentiating cells modifies the expression of markers of specific neuronal subtypes, increasing the expression of cholinergic, dopaminergic and GABAergic markers. These results may be consistent with the observed effect of mild hypoxia on neural progenitor cell differentiation towards a dopaminergic phenotype [41] or with the effects of pollutants that increase intracellular ROS levels on the maturation of GABAergic neurons [42]. Less prominent are the effects observed on the expression of glutamatergic or serotoninergic markers. Intriguingly, although NT2-D1 cells may generate rare serotoninergic neurons [43], we observed that SERT expression peaked at a relatively early time-point (4 weeks) and significantly decreased after 2 additional weeks of culture. This event may be a consequence of the mechanical selection of cells that is performed after 4 weeks of differentiation. However, the addition of E3330 did not affect SERT expression levels at earliest time point.

In conclusion, we have demonstrated that APE1 is involved in the differentiation of adult and embryonic stem cells towards a neuronal fate. The inhibition of APE1 redox function, through the use of the specific inhibitor E3330, demonstrates that this factor may act by both repressing neuronal maturation and biasing the differentiation towards specific neuronal subtypes. The majority of the transcription factors that are regulated by APE1 in a redox-dependent fashion act mainly favoring progenitor cell proliferation and differentiation, while p53 and STAT3 exert a repressive effect on neurogenesis. E3330 may act either inhibiting these latter factors or shifting stimuli from proliferation to differentiation. Therefore, this work emphasizes the possible use of pharmacological strategies, based on modulation of APE1 redox functions, to boost neural differentiation and bias the differentiation potential of stem cells towards specific neuronal subtypes. In this regard, the possibility to employ a small molecule, such as E3330, to increase the extent of differentiation of stem cells towards a dopaminergic phenotype is a very attractive one.

## Supporting Information

Figure S1 **Immunofluorescence images of undifferentiated NT2-D1 cells (upper panels) and NT2-D1 cells exposed for 1 week to ATRA (lower panels).** Typical markers of undifferentiated cells Nanog (purple), Nestin (green) and Sox2 (yellow) were tested. Histograms comprised in the mid box represent the fraction (%) of cells strongly positive to the tested markers. Histograms contained in the low box show the fraction of cells either strongly or weakly positive to Sox2.
(TIF)

Figure S2 **Scheme showing the markers chosen to identify neural precursors, glial and neuronal subtypes.**
(TIF)

Table S1 **Primary and secondary antibodies utilized. RP = rabbit polyclonal; MM = mouse monoclonal.**
(DOCX)

Table S2 **Real-Time PCR primers.**
(DOCX)
