# Supplementary figures and images for: The Redox Function of APE1 Is Involved in the Differentiation Process of Stem Cells toward a Neuronal Cell Fate
Source: PLoS One. 2014 Feb 19;9(2):e89232. doi: 10.1371/journal.pone.0089232 (PMC3929656; doi:10.1371/journal.pone.0089232)

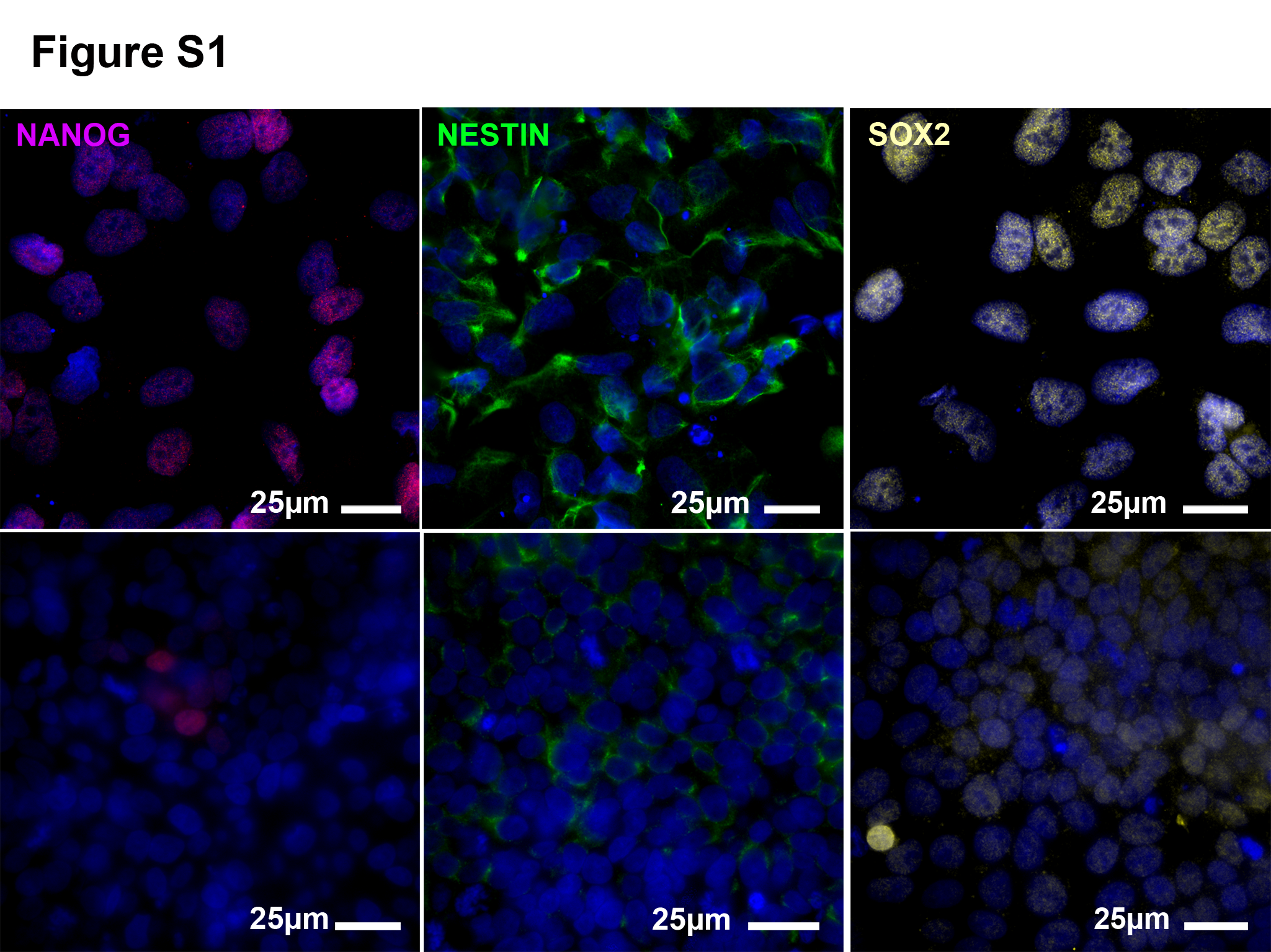

Supplement: Figure S1 — Immunofluorescence images of undifferentiated NT2-D1 cells (upper panels) and NT2-D1 cells exposed for 1 week to ATRA (lower panels). Typical markers of undifferentiated cells Nanog (purple), Nestin (green) and Sox2 (yellow) were tested. Histograms comprised in the mid box represent the fraction (%) of cells strongly positive to the tested markers. Histograms contained in the low box show the fraction of cells either strongly or weakly positive to Sox2. (TIF) [file pone.0089232.s001.tif]

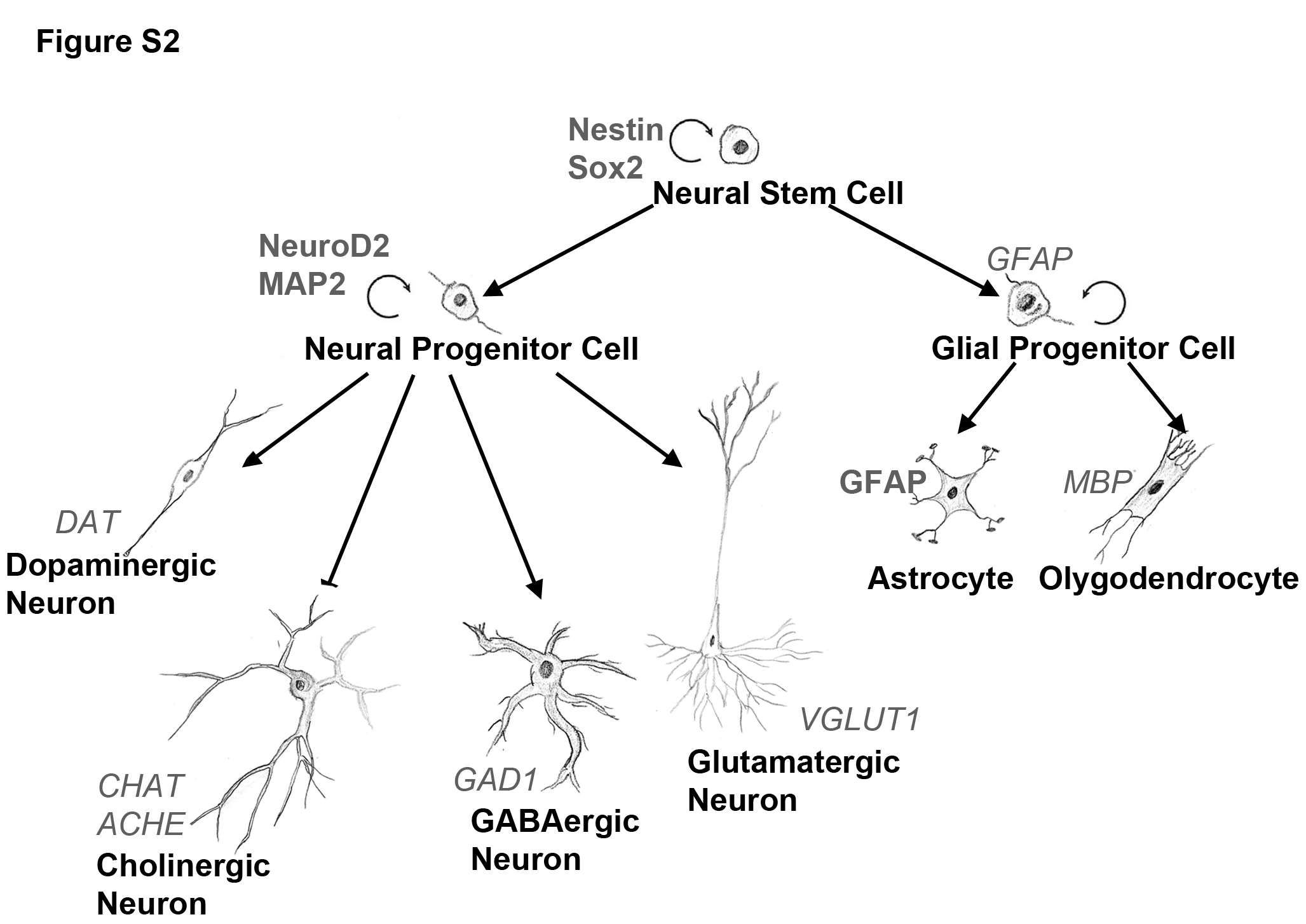

Supplement: Figure S2 — Scheme showing the markers chosen to identify neural precursors, glial and neuronal subtypes. (TIF) [file pone.0089232.s002.tif]
