# Supplementary material for: The Redox Function of APE1 Is Involved in the Differentiation Process of Stem Cells toward a Neuronal Cell Fate
Source: PLoS One. 2014 Feb 19;9(2):e89232. doi: 10.1371/journal.pone.0089232 (PMC3929656; doi:10.1371/journal.pone.0089232)
Supplement: Table S1 — Primary and secondary antibodies utilized. RP = rabbit polyclonal; MM = mouse monoclonal. (DOCX) [file pone.0089232.s003.docx]

| **PRIMARY ANTIBODY** | | | | **SECONDARY ANTIBODY** | | |
| --- | --- | --- | --- | --- | --- | --- |
| **Antigen** | **Producer** | **Permeabilization protocol** | **Dilution** | **Fluorochrome** | **Producer** | **Dilution** |
| **OCT4** | ABCAM. RP | TRITON X-100 0.1% | 1:200 | A488 | Molecular Probes | 1:400 |
| **NANOG** | ABCAM. RP | TRITON X-100 0.1% | 1:200 | A488 | Molecular Probes | 1:400 |
| **SOX2** | ABCAM. RP | TRITON X-100 0.1% | 1:200 | A488 | Molecular Probes | 1:400 |
| **GATA4** | S. CRUZ. RP | TRITON X-100 0.1% | 1:50 | A488 | Molecular Probes | 1:400 |
| **CK 8-18-19** | BIOGENEX. MM | TRITON X-100 0.1% | 1:20 | A555 | Molecular Probes | 1:800 |
| **SMA** | DAKO. MM | TRITON X-100 0.1% | 1:50 | A488 | Molecular Probes | 1:400 |
| **CONNEXIN 43** | S. CRUZ. RP | / | 1:40 | A488 | Molecular Probes | 1:400 |
| **ASA** | SIGMA. MM | TRITON X-100 0.1% | 1:100 | A555 | Molecular Probes | 1:800 |
| **GFAP** | DAKO. RP | TRITON X-100 0.1% | 1:50 | A488 | Molecular Probes | 1:400 |
| **NSE** | DAKO. MM | TRITON X-100 0.1% | 1:50 | A488 | Molecular Probes | 1:400 |
| **B3 TUBULIN** | ABCAM. MM | TRITON X-100 0.1% | 1:1000 | A555 | Molecular Probes | 1:800 |
| **AchT** | ABCAM. | Antigen Retrieval Buffer (Dako), 40’ at 98 °C | 1:2000 | Envision | DAKO |  |

Table S1. Primary and secondary antibody utilized. RP=rabbit polyclonal; MM=mouse monoclonal.
