# Supplementary material for: The Redox Function of APE1 Is Involved in the Differentiation Process of Stem Cells toward a Neuronal Cell Fate
Source: PLoS One. 2014 Feb 19;9(2):e89232. doi: 10.1371/journal.pone.0089232 (PMC3929656; doi:10.1371/journal.pone.0089232)
Supplement: Table S2 — Real-Time PCR primers. (DOCX) [file pone.0089232.s004.docx]

**Table S2. Real-Time PCR primers.**

| **GENE** | **FORWARD PRIMER** | **REVERSE PRIMER** |
| --- | --- | --- |
| **ACHE** | CATCAACGCGGGAGACTT | GAGACTCGTTGTCTTTGCTGAA |
| **CHAT** | CCCTGATGCCTTCATCCA | GTAGGTGGGCACCAGTCTTC |
| **DAT** | ATTGGCTTTGCTGTGGACCT | AAGTGGCATCCCAGCAATGA |
| **GAD1** | TCAAGTAAAGATGGTGATGGGATA | GCCATGATGCTGTACATGTTG |
| **GFAP** | CCAACCTGCAGATTCGAGA | TCTTGAGGTGGCCTTCTGAC |
| **GRIN2A** | CCGAGAAGGCCAGTACGA | GTTGTGGCAGATCCCAGTG |
| **MAP2** | TCACCTGCCTCAGAACAGACT | ACTGGGAGCCAGAGCTGAT |
| **MBP 75** | CCGAGAAGGCCAGTACGA | GTGAAAGTTCACCCAGGTTTCT |
| **MBP 116** | AAAGGTTCAAGCGTCTTTGG | CCTGAATGGATTGGCTCTTC |
| **NSE** | TTGTCAGGGACTATCCTGTGG | TCCCTACATTGGCTGTGAACT |
| **NEUROD2** | GGGGCAGAAGGAGGTGAG | CCCAGGAGGGGACAGAAT |
| **SERT** | TCACAGTGCTCGGTTACATGGC | GAAAGTGGACGCTGGCATGTTG |
| **VGLUT1** | TTTTCTGGGGCTACATTGTCAC | ACTCCGTTCTAAGGGTGGGG |
| **HPRT** | GACCAGTCAACAACAGGGGACAT | GTGTCAATTATATCTTCCACAATCAAG |
